# Inspiratory muscle warm-up has no impact on performance or locomotor muscle oxygenation during high-intensity intermittent sprint cycling exercise

**DOI:** 10.1186/s40064-015-1355-2

**Published:** 2015-09-28

**Authors:** Toshiyuki Ohya, Masahiro Hagiwara, Yasuhiro Suzuki

**Affiliations:** Department of Sports Science, Japan Institute of Sports Sciences, 3-15-1 Nishigaoka, Kita-ku, Tokyo, 115-0056 Japan

**Keywords:** Near-infrared spectroscopy, Repeated-sprint, Team sports, Respiratory muscle, Inspiratory muscle fatigue

## Abstract

The purpose of this study was to investigate the effect of inspiratory muscle (IM) warm-up on performance and locomotor muscle oxygenation during high-intensity intermittent sprint cycling exercise. Ten subjects performed identical exercise tests (10 × 5 s with 25-s recovery on a cycle ergometer) after performing one of two different IM warm-up protocols. The IM warm-up consisted of two sets of 30 inspiratory efforts against a pressure-threshold load equivalent to 15 % (PLA) or 40 % (IMW) of maximal inspiratory pressure (MIP). MIP was measured with a portable autospirometer. Peak power and percent decrease in power were determined. Oxyhemoglobin (O_2_Hb) was measured using near-infrared spectroscopy. The MIP increased relative to baseline after IMW (115 ± 21 vs. 123 ± 17 cmH_2_O, *P* = 0.012, ES = 0.42), but not after PLA (115 ± 20 vs. 116 ± 17 cmH_2_O). Peak power (PLA: 10.0 ± 0.6 vs. IMW: 10.2 ± 0.5 W kg^−1^), percent decrease in power (PLA: 13.4 ± 5.6 vs. IMW: 13.2 ± 5.5 %), and changes in O_2_Hb levels (PLA: −10.8 ± 4.8 vs. −10.7 ± 4.1 μM) did not differ between the trials. IM function was improved by IMW. However, this did not enhance performance or locomotor muscle oxygenation during high-intensity intermittent sprint cycling exercise in untrained healthy males.

## Background

The ability to repeat high-intensity short-duration sprints is an important component of fitness for field-based team sports (Mohr et al. 2003) such as soccer, rugby, and field hockey. A warm-up is defined as any preliminary performance that enhances physical activity (Bishop et al. 2001; Burnley et al. 2005). Warming up has been shown to improve performance during single sprints and during the first in a series of intermittent sprints (Yaicharoen et al. 2012). Thus, optimal warm-up is essential for optimal high-intensity exercise performance.

It was recently shown that inspiratory muscle (IM) warm-up, in addition to whole-body warm-up, improves exercise performance. IM warm-up appears to improve IM function (Lin et al. 2007; Lomax et al. 2011; Tong and Fu 2006; Volianitis et al. 2001), reduce the perception of breathlessness (Tong and Fu 2006; Volianitis et al. 2001), and result in lower lactate concentrations during exercise (Lin et al. 2007). An IM warm-up protocol performed at 40 % of maximal inspiratory pressure (MIP) improved badminton footwork performance (Lin et al. 2007) and intermittent running performance (Lomax et al. 2011). Cheng et al. (2013) also reported less reduction in the saturation index of the leg muscles, assessed by near-infrared spectroscopy (NIRS), during two 6-min submaximal cycling exercise sessions followed by high-intensity intermittent sprint tests (6 × 10 s with 60-s recovery) with IM warm-up than without IM warm-up. Although high-intensity intermittent performance was not improved by IM warm-up in that trial, the IM warm-ups were not conducted immediately before the high-intensity intermittent sprint test (Cheng et al. 2013). Thus, the actual effect of IM warm-up on high-intensity intermittent exercise performance remains unclear.

Maintenance of power output during high-intensity intermittent exercise is thought to be limited by depletion of phosphocreatine (PCr) stores (Gaitanos et al. 1993). An accelerated drop in accessory respiratory muscle oxygenation was related to an attenuated fall in leg muscle oxygenation in heavy exercise (Legrand et al. 2007). Thus, the high oxygen requirement of respiratory muscle may lead to limited oxygen use by locomotor muscles (Legrand et al. 2007). Additionally, respiratory muscle fatigue appears to result from increased respiratory muscle work combined with competition with the locomotor muscles of the limbs for blood flow (Romer and Polkey 2008). Previous studies (Lin et al. 2007; Tong and Fu 2006; Volianitis et al. 2001) have suggested that improved performance following IM warm-up is partly attributable to enhanced IM function. Improved IM function might delay IM fatigue, thus influencing the supply and delivery of oxygen to working locomotor muscles. Indeed, high-intensity intermittent cycling exercise performance was related to changes in locomotor muscle oxygenation (Ohya et al. 2013). As PCr resynthesis depends on oxygen availability (Harris et al. 1976; Haseler et al. 1999), inhibition of IM fatigue might contribute to maintenance of performance during high-intensity intermittent sprint exercise.

In the present study, we investigated the effect of IM warm-up on performance and muscle oxygenation during high-intensity intermittent sprint cycling exercise. We hypothesized that IM warm-up would improve high-intensity intermittent sprint cycling exercise performance by enhancing IM function and attenuating locomotor muscle deoxygenation during exercise. To test this hypothesis, we used 10 repeated maximal 5-s sprints that were alternated with 25 s of active recovery under two different IM warm-up conditions. This intermittent exercise performance was related to changes in locomotor muscle oxygenation (Ohya et al. 2013).

## Methods

### Subjects

Ten healthy males participated in this study (mean ± SD: age, 25.1 ± 4.8 years; height, 172.4 ± 7.2 cm; body mass, 64.1 ± 10.8 kg). All subjects were involved in different physical activities such as soccer, cycling, swimming, and running. Weekly physical activities typically consisted of three sessions (30–60 min per session). No subjects had any history or clinical signs of cardiovascular or pulmonary disease. Written voluntary consent to participate was obtained from all subjects after informing them of the purpose of the experiment, the procedure, and the possible risks. This study was approved by the Human Subjects Committee at the Japan Institute of Sports Sciences.

### Experimental overview

Subjects visited the laboratory four times. During the first visit, they completed an assessment of dynamic pulmonary function and were familiarized with the MIP and sprint cycling tests. During the second visit, a maximal graded exercise test was performed. During the third and fourth visits, which were separated by at least 48 h, 10 repeated maximal 5-s sprints were alternated with 25 s of active recovery at 40 % of peak oxygen uptake (${\dot{\text{V}}}$O_2_peak) on a cycle ergometer (Powermax-V II; Combi Wellness, Tokyo, Japan) under two different IM warm-up conditions. All experimental trials were conducted at the same time of day (±1 h) for each subject to minimize the effect of diurnal variation. Subjects were instructed to grip the handlebars and to remain seated while cycling, and all subjects were familiarized with sprinting on the cycle ergometer before the study. The subjects were required to consume their last meal at least 3 h prior and to refrain from drinking caffeinated beverages at least 10 h prior to the test.

### Maximal graded exercise test

The maximal graded exercise test was performed on the cycle ergometer to determine ${\dot{\text{V}}}$O_2_peak. After a 5-min warm-up at 75 W, the power output was increased by 30 W every 2 min until exhaustion. The subjects were asked to maintain a cadence of 75 rpm during this test (Ohya et al. 2013). The test was terminated when they could not maintain the required cadence despite vigorous encouragement (the range of maximal graded exercise test durations was from 14 to 20 min, and the mean duration was 17 min in this study).

### Inspiratory muscle (IM) warm-up

The IM warm-up protocol consisted of two sets of 30 breaths using a POWERbreathe IM trainer (IMT Technologies Ltd., Birmingham, UK) with an inspiratory-pressure threshold load equivalent to 15 % (PLA) and 40 % (IMW) of MIP, with a 60-s rest between sets (Lomax et al. 2011). During the IMW trial, the subjects were instructed to initiate every breath from the residual volume and to continue the respiratory effort up to the lung volume where the IM force output for the given load limited further excursion of the thorax. During the PLA trial, the breaths were performed gently and the respiratory time for each breath was protracted. The subjects were blinded to the true purpose of the study and were told that they were participating in a study comparing the effects of powerful-type (IMW) and endurance-type (PLA) IM warm-up protocols on subsequent intermittent sprint cycling performance. The IM warm-up was undertaken while seated with the nose occluded.

### Intermittent sprint cycling exercise tests

A standardized whole-body warm-up was performed prior to two intermittent sprint cycling exercise tests. The whole-body warm-up comprised 5 min of stretching exercise and 5 min of cycling at 40 % ${\dot{\text{V}}}$O_2_peak, including a 3-s maximal sprint. The IM warm-up condition (IMW or PLA) was randomly assigned and was performed between the stretching exercise and the cycling warm-up. MIP was measured after the cycling warm-up, followed by 3 min passive rest seated on the saddle, then the testing commenced. The intermittent sprint cycling exercise tests were ten 5-s maximal sprints separated by 25 s of active recovery (40 % ${\dot{\text{V}}}$O_2_peak). In all trials, the subjects were required to assume the start position at 5 s before the start of the next sprint. The braking force adjusted corresponding to 75 g kg^−1^ of their body mass.

Handlebars and saddle height were adjusted according to the preference of the subject and were kept constant for each trial. Strong verbal encouragement was provided to each subject during all sprints. Peak power was defined as the highest power reading at each sprint, calculated using the original test program. The percent decrease in peak power, which was used to assess fatigue during the test, was calculated as follows: 100 − [(total power/ideal power) × 100]. Total power was sum of peak power values from all sprints. Ideal power was peak power of the first bout multiplied by 10 (Fitzsimons et al. 1993). The ideal power was calculated separately for each subject in each trial. This has previously been shown to be the most reliable method of assessing fatigue during intermittent sprint tests (Glaister et al. 2004).

### Measurements

#### Maximal inspiratory pressure (MIP)

MIP, which is commonly used to measure inspiratory muscle strength, was assessed according to published guidelines (American Thoracic Society/European Respiratory Society 2002). MIP was measured before stretching exercise, after cycling warm-up, and within 2 min after the high-intensity intermittent sprint cycling exercise using a portable autospirometer (AS-507; Minato Medical Science, Osaka, Japan) with a handheld mouth pressure meter (AAM377; Minato Medical Science) (Katayama et al. 2012). All measurements were made with the participant in a sitting position with the nose occluded. Participants were instructed to breathe out to residual volume, then inhale as hard and as quickly as possible to total lung capacity and sustain this inspiration for at least 1 s. Measurements were repeated until a minimum of five and a maximum of seven technically satisfactory measurements were obtained, and the greatest of the three measurements that had less than 5 % variability or that were within 5 cmH_2_O of each other was defined as the maximum (Wen et al. 1997).

#### Pulmonary function

Pulmonary function was assessed using a portable autospirometer (AS-507; Minato Medical Science) (Katayama et al. 2012) to determine forced vital capacity (FVC) and forced expiratory volume in 1 s (FEV_1_). All measurements were begun at total lung capacity with participants in a sitting position with the nose occluded, and were performed according to published guidelines (Miller et al. 2005).

#### Near-infrared spectroscopy

Changes in tissue oxyhemoglobin (O_2_Hb) and deoxyhemoglobin (HHb) were estimated during the intermittent sprint cycling exercise tests using a NIRS device (NIRO-200; Hamamatsu Photonics K.K., Shizuoka, Japan), as previously described (Buchheit et al. 2009; Katayama et al. 2010). The device consists of a continuous-wave near-infrared spectrophotometer that generates a light source using 775-, 810-, and 850-nm wavelengths. The changes in absorption of O_2_Hb and HHb were determined using the modified Lambert–Beer law. To record the NIRS signal, the probe was placed over the right vastus lateralis muscle, 20–24 cm proximal to the knee along the vertical axis of the thigh. The detector in the NIRS probe was separated from the light source by 40 mm. An indelible marker was used to mark the position of the optodes to ensure correct and consistent placement during the intermittent sprint cycling exercise tests. The optodes and skin were covered with a black rubber holder and secured with double-sided surgical tape to prevent contamination by ambient light.

Subcutaneous fat thickness was measured before the tests using ultrasound (SSD-900; Hitachi-Aloka Medical, Tokyo, Japan) to determine the optimal optode position; the combined thickness of the skin and subcutaneous fat must be less than half the distance between the source and the detector to allow the NIRS photons to penetrate through to the muscle. Data were sampled at 5 Hz. The O_2_Hb and HHb values before the 2-min intermittent sprint cycling exercise test were defined as zero. The NIRS signal was analyzed across a 1-s period. The variation in O_2_Hb corresponded to the difference between the lowest values and the highest values during each recovery period (i.e., muscle Δoxygenation, O_2_HbΔ). The variation in HHb corresponded to the difference between the highest values and the lowest values during each recovery period.

#### Cardiorespiratory measurements

Breath-by-breath respiratory gas exchange was measured using an automated gas analysis system (AE-310 s; Minato Medical Science) to determine ventilation (${\dot{\text{V}}}$E), oxygen uptake (${\dot{\text{V}}}$O_2_), and carbon dioxide production (${\dot{\text{V}}}$CO_2_) during all exercise tests. Respiratory gas exchange values were averaged every 30 s for the maximal graded exercise test and every 5 s for the intermittent sprint cycling exercise tests. Heart rate (HR) (RS800; Polar Electro, Kempele, Finland) was averaged every 30 s for the maximal graded exercise test. The gas analysis system was calibrated using a gas mixture of known O_2_ and CO_2_ concentrations before each test. The volume transducer was calibrated before each test using a 2-L syringe (Minato Medical Science). Maximal values were recorded for the maximal graded exercise test, whereas mean values were recorded for the intermittent sprint cycling exercise tests. The ${\dot{\text{V}}}$O_2_peak and maximal ${\dot{\text{V}}}$E (${\dot{\text{V}}}$Emax) were defined as the highest ${\dot{\text{V}}}$O_2_ and ${\dot{\text{V}}}$E attained during the maximal graded exercise test.

### Blood lactate concentration

At 1 and 3 min after the end of the maximal graded exercise test and intermittent sprint cycling exercise tests, fingertip blood samples (0.3 μl) were collected to measure blood lactate concentrations ([La]_b_) using the enzymatic-amperometric detection method (Lactate Pro 2LT-1730; Arkray, Kyoto, Japan). The peak [La]_b_ value was recorded as the highest value obtained after the end of the tests.

### Statistical analysis

Data are expressed as mean ± SD. Statistical analyses were performed using IBM SPSS Statistics for Windows, Version 19.0 (IBM Corp., Armonk, NY, USA). Analysis of the MIP (IM warm-up condition and measured point) and peak power values (IM warm-up condition and sprint repetition) from the intermittent sprint cycling exercise tests were conducted using two-way analysis of variance (ANOVA) with repeated measures. Significant interactions were then assessed using Bonferroni post hoc tests. Paired *t*-tests were used to assess the differences in peak power decrement, peak [La]_b_, O_2_HbΔ, HHbΔ, and cardiorespiratory measurements from the intermittent sprint cycling exercise tests. Significance was set at *P* < 0.05.

## Results

### Maximal graded exercise test

The mean values obtained for the maximal graded exercise test were ${\dot{\text{V}}}$O_2_peak 44.5 ± 5.8 ml kg^−1^ min^−1^, ${\dot{\text{V}}}$Emax 148.7 ± 17.0 l min^−1^, HRmax 185 ± 12 beats min^−1^, and peak [La]_b_ 14.0 ± 1.6 mmol l^−1^.

#### Maximal inspiratory pressure and pulmonary function

**Fig. 1:**
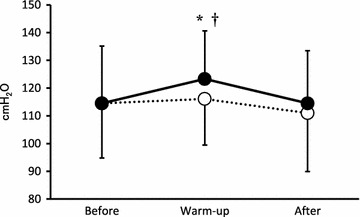
Maximal inspiratory pressure (MIP) before stretching exercise (*Before*), after inspiratory muscle (IM) warm-up (*Warm-up*), and after the intermittent sprint cycling exercise tests (*After*) for two different IM warm-up conditions [warm-up at 15 % (PLA: *unfilled circle*) and 40 % (IMW: *filled circle*) of MIP]. Values are mean ± SD (n = 10). * Significant difference vs. PLA (*P* < 0.05). ^†^ Significant difference vs. before IM warm-up (*P* < 0.05)

MIP values before stretching exercise, after IM warm-up, and after the intermittent sprint cycling exercise tests are shown in Fig. 1. The MIP values before stretching exercise did not significantly differ (PLA: 115 ± 20 vs. IMW: 115 ± 21 cmH_2_O). For the IMW condition, the mean MIP after IM warm-up was higher than before stretching exercise [IMW; before: 115 ± 21 vs. after: 123 ± 17 cmH_2_O, *P* = 0.012, Effect size (ES) = 0.42] and higher than the PLA value after IM warm-up (PLA: 116 ± 17 vs. IMW: 123 ± 17 cmH_2_O, *P* = 0.005, ES = 0.41). The MIP values after the intermittent sprint cycling exercise tests were not significantly different between conditions (PLA: 111 ± 21 vs. IMW: 115 ± 19 cmH_2_O). Mean FVC and FEV_1_ were 4.47 ± 0.37 and 3.96 ± 0.28 l, respectively.

### Intermittent sprint cycling exercise tests

#### Peak power

**Fig. 2:**
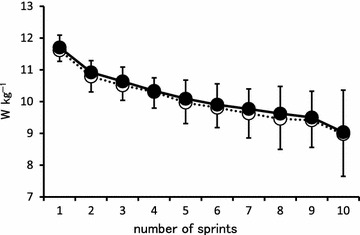
Peak power values during the intermittent sprint cycling exercise tests for two different inspiratory muscle warm-up conditions [warm-up at 15 % (PLA: *unfilled circle*) and 40 % (IMW: *filled circle*) of maximal inspiratory pressure]. Values are mean ± SD (n = 10)

The peak power values for the intermittent sprint cycling exercise tests are shown in Fig. 2. The mean peak power values were not significantly different between conditions (PLA: 10.0 ± 0.6 vs. IMW: 10.2 ± 0.5 W kg^−1^). The percent decrease in peak power was not significantly different between conditions (PLA: 13.4 ± 5.6 vs. IMW: 13.2 ± 5.5 %).

#### Near-infrared spectroscopy

**Fig. 3:**
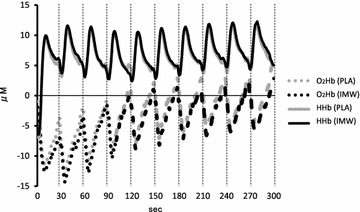
Time-course of mean oxyhemoglobin (O_2_Hb: *dashed line*) and deoxyhemoglobin (HHb: *line*) concentrations during the intermittent sprint cycling exercise tests for two different inspiratory muscle warm-up conditions [warm-up at 15 % (PLA) and 40 % (IMW) of maximal inspiratory pressure] (n = 10)

The changes in O_2_Hb and HHb measured throughout the intermittent sprint cycling exercise tests for PLA vs. IMW are shown in Fig. 3. The changes in O_2_Hb and HHb during the recovery period between the intermittent sprint cycling exercise tests are shown in Table 1. The mean values of O_2_HbΔ and HHbΔ during the recovery period between the intermittent sprint cycling exercise tests did not significantly differ between IM warm-up conditions.

**Table 1:** The mean of the individual changes in oxyhemoglobin (O_2_HbΔ) and deoxyhemoglobin (HHbΔ) during the nine recovery periods between the intermittent sprint cycling exercise tests following inspiratory muscle warm-up at 15 % (PLA) or 40 % (IMW) of maximal inspiratory pressure

	PLA	IMW
O_2_HbΔ (μM)	−10.8 ± 4.8	−10.7 ± 4.1
HHbΔ (μM)	8.0 ± 3.7	8.7 ± 3.7

Values are expressed as mean ± SD (n = 10)

#### Cardiorespiratory measurements and blood lactate concentrations

The mean ${\dot{\text{V}}}$E, ${\dot{\text{V}}}$O_2_, ${\dot{\text{V}}}$CO_2_, and peak post-exercise [La]_b_ values during the intermittent sprint cycling exercise tests are shown in Table 2. The mean ${\dot{\text{V}}}$E, ${\dot{\text{V}}}$O_2_, ${\dot{\text{V}}}$CO_2_, and peak post-exercise [La]_b_ values did not significantly differ between IM warm-up conditions.

**Table 2:** Cardiorespiratory measurements and peak post-exercise blood lactate concentrations (La) during intermittent sprint cycling exercise tests following inspiratory muscle warm-up at 15 % (PLA) or 40 % (IMW) of maximal inspiratory pressure

	PLA	IMW
${\dot{\text{V}}}$E (l min^−1^)	82.9 ± 17.1	83.9 ± 17.7
${\dot{\text{V}}}$O_2_ (ml kg^−1^ min^−1^)	33.0 ± 3.7	32.4 ± 4.1
${\dot{\text{V}}}$CO_2_ (ml kg^−1^ min^−1^)	37.1 ± 3.7	37.4 ± 3.9
La (mmol l^−1^)	9.8 ± 2.7	10.2 ± 2.9

Values are expressed as mean ± SD (n = 10)

## Discussion

The aim of this study was to investigate the effects of IM warm-up on performance and muscle oxygenation during high-intensity intermittent sprint cycling exercise. The major findings of this study were: (1) MIP values were significantly higher after IM warm-up under IMW conditions than with PLA conditions; (2) peak power and percent decrease in peak power did not differ between IM warm-up conditions; and (3) mean O_2_HbΔ was not different between IM warm-up conditions. We hypothesized that IMW would positively influence high-intensity intermittent sprint cycling exercise performance by delaying IM fatigue and thereby attenuating the muscle deoxygenation that occurs during intense exercise.

Several studies have shown that an IM warm-up consisting of two sets of 30 breaths at 40 % of MIP resulted in improved MIP (Lin et al. 2007; Lomax et al. 2011; Tong and Fu 2006; Volianitis et al. 2001). Similarly, in the present study, MIP values were improved after IM warm-up under IMW conditions (Fig. 1). These changes were previously attributed to improved IM coordination and increased voluntary activation of IM (Hawkes et al. 2007; Ross et al. 2007). Further, it was suggested that increases in MIP reduces the fractional use of maximum tension generated with each inspiration.

The peak power values and the percent decrease in peak power during the intermittent sprint cycling exercise tests did not significantly differ between conditions (Fig. 2). These findings are inconsistent with the results of previous studies (Lin et al. 2007; Lomax et al. 2011; Tong and Fu 2006), in which IM warm-up enhanced intermittent exercise performance. A possible explanation for these discrepant findings may relate to the exercise modality used. Previous studies that demonstrated a positive effect of IM warm-up on intermittent exercise performance used incremental running to exhaustion as the exercise. Intra-abdominal pressure is increased during running, which is thought to stabilize the spine (Grillner et al. 1978). The diaphragm is also activated to increase intra-abdominal pressure during movement of the upper limbs, such as that which occurs during running (Hodges et al. 1997; Hodges and Gandevia 2000). In previous studies using running exercise, increased diaphragmatic activation may have induced earlier IM fatigue, which was mitigated by IM warm-up, thus improving performance.

The enhanced performance induced by the IM warm-up protocol in previous studies may also relate, at least in part, due to a reduction in the sensation of breathlessness (Lin et al. 2007; Tong and Fu 2006). Volianitis et al. (2001) suggested that IM fatigue was related to the perception of dyspnea. Because IM fatigue (defined as a fall in MIP) did not occur during the short-duration intermittent sprint cycling exercise tests (ten 5-s maximal sprints separated by 25 s of active recovery) in the present study (Fig. 1), the participants might not have felt a sensation of breathlessness and, thus, IM warm-up might not have affected their performance. Similarly, IM warm-up was found to have no effect on high-intensity intermittent cycling exercise performance (six 10-s maximal cycling sprints separated by 60 s of active recovery), although post-exercise MIP was not assessed (Cheng et al. 2013).

Exercise-induced IM fatigue is affected by exercise modality as well as exercise intensity and duration. Johnson et al. (1993) argued that the magnitude of diaphragmatic fatigue and the likelihood of its occurrence increases as the relative intensity of the exercise exceeds 85 % ${\dot{\text{V}}}$O_2_max. Several studies have reported that exercising to exhaustion (8–10 min) at intensities of at least 80–85 % ${\dot{\text{V}}}$O_2_max induced 15–30 % reductions in transdiaphragmatic pressure (Babcock et al. 1996; Babcock et al. 2002; Johnson et al. 1993; Romer and Polkey 2008). By contrast, short-term incremental exercise to exhaustion did not affect transdiaphragmatic pressure (Romer et al. 2007). In the present study, the lack of IM fatigue during the high-intensity intermittent sprint cycling exercise tests may relate to the short duration of exercise used (50 s). Further studies are required to establish the effect of an IM warm-up on performance during a prolonged session of repeated short-duration intermittent cycling sprints, which can induce extreme IM fatigue.

Respiratory muscle fatigue appears to result from increased respiratory muscle work combined with competition with the locomotor muscles of the limbs for blood flow (Romer and Polkey 2008). Additionally, respiratory muscle fatigue can increase sympathetic vasoconstrictor outflow to the working skeletal muscles through a respiratory muscle metaboreflex (Katayama et al. 2012). Legrand et al. (2007) reported that an accelerated drop in accessory respiratory muscle oxygenation was related to the attenuated fall in leg muscle oxygenation detected with NIRS in heavy exercise. These data suggest that the high oxygen requirement of respiratory muscle leads to limited oxygen use by locomotor muscles (Legrand et al. 2007). Thus, IM work might limit oxygen use by the locomotor muscles (Katayama et al. 2012; Legrand et al. 2007; Romer and Polkey 2008).

The mean O_2_HbΔ did not significantly differ between conditions in the present study (Table 1). We previously reported that high-intensity intermittent cycling exercise performance was related to changes in locomotor muscle oxygenation (Ohya et al. 2013). Depletion of PCr stores limits performance during high-intensity intermittent exercise (Gaitanos et al. 1993). Because PCr resynthesis requires oxygen (Harris et al. 1976; Haseler et al. 1999), we considered that inhibition of IM fatigue through IM warm-up exercises would improve performance during the high-intensity intermittent sprint cycling exercise. However, IMW did not influence exercise performance, and may not have influenced muscle oxygen availability during this study. As IM fatigue did not occur during the high-intensity intermittent sprint cycling exercise tests in this study, oxygen availability might not have been affected regardless of IM treatment.

Cheng et al. (2013) reported an improvement in leg muscle saturation index during two 6-min submaximal cycling exercise sessions followed by high-intensity intermittent sprint tests (6 × 10 s with 60-s recovery) with IM warm-up in females. However, IM warm-up did not enhance locomotor muscle oxygenation in males in this study. Thus, differences in sex may partly explain these discrepancies, as there was no improvement in leg muscle saturation index by IM warm-up in males. Further, repeated sprints exercise induced a smaller reduction of glycogen in type I fibers in females compared with males (Esbjörnsson-Liljedahl et al. 2002), suggesting that oxidative capacity in locomotor muscles might be relatively higher in females than in males (Cheng et al. 2013).

There are two potential limitations of our study. First, the inspiratory muscle strength measurements were taken using volitional, non-invasive techniques, which are better tolerated by participants than the use of balloon catheter systems. Although non-invasive, these techniques are reliable and valid for the measurement of inspiratory muscle strength (Romer and McConnell 2004; Wijkstra et al. 1995). Another limitation of this study was that we only measured locomotor muscle oxygenation, and did not measure the oxygenation of the inspiratory muscles. Although the locomotor muscle oxygenation was not significantly different between the IM warm-up conditions in our study, there may have been an enhancement of oxygenation of the IM during exercise as a result of the IM warm-up. Further studies are required to clarify the effects of IM warm-up conditions on IM oxygenation.

In conclusion, we found that IM-specific warm-up improves IM function. However, IM warm-up did not improve high-intensity intermittent sprint cycling exercise performance or locomotor muscle oxygenation during exercise in untrained healthy males. These results suggest that IM warm-up may not influence performance during exercise, such as high-intensity intermittent sprint cycling.

## References

[CR1] American Thoracic Society/European Respiratory Society (2002). ATS/ERS statement on respiratory muscle testing. Am J Respir Crit Care Med.

[CR2] Babcock MA, Pegelow DF, Johnson BD, Dempsey JA (1996). Aerobic fitness effects on exercise-induced low-frequency diaphragm fatigue. J Appl Physiol.

[CR3] Babcock MA, Pegelow DF, Harms CA, Dempsey JA (2002). Effects of respiratory muscle unloading on exercise-induced diaphragm fatigue. J Appl Physiol.

[CR4] Bishop D, Bonetti D, Dawson B (2001). The effect of three different warm-up intensities on kayak ergometer performance. Med Sci Sports Exerc.

[CR5] Buchheit M, Cormie P, Abbiss CR, Ahmaidi S, Nosaka KK, Laursen PB (2009). Muscle deoxygenation during repeated sprint running: effect of active vs. passive recovery. Int J Sports Med.

[CR6] Burnley M, Doust JH, Jones AM (2005). Effects of prior warm-up regime on severe-intensity cycling performance. Med Sci Sports Exerc.

[CR7] Cheng CF, Tong TK, Kuo YC, Chen PH, Huang HW, Lee CL (2013). Inspiratory muscle warm-up attenuates muscle deoxygenation during cycling exercise in women athletes. Respir Physiol Neurobiol.

[CR8] Esbjörnsson-Liljedahl M, Bodin K, Jansson E (2002). Smaller muscle ATP reduction in women than in men by repeated bouts of sprint exercise. J Appl Physiol.

[CR9] Fitzsimons M, Dawson B, Ward D, Wilkinson A (1993). Cycling and running test of repeated sprint ability. Aust J Sci Med Sports.

[CR10] Gaitanos GC, Williams C, Boobis LH, Brooks S (1993). Human muscle metabolism during intermittent maximal exercise. J Appl Physiol.

[CR11] Glaister M, Stone MH, Stewart AM, Hughes M, Moir GL (2004). The reliability and validity of fatigue measures during short-duration maximal-intensity intermittent cycling. J Strength Cond Res.

[CR12] Grillner S, Nilsson J, Thorstensson A (1978). Intra-abdominal pressure changes during natural movements in man. Acta Physiol Scand.

[CR13] Harris RC, Edwards RH, Hultman E, Nordesjö LO, Nylind B, Sahlin K (1976). The time course of phosphorylcreatine resynthesis during recovery of the quadriceps muscle in man. Pflugers Arch.

[CR14] Haseler LJ, Hogan MC, Richardson RS (1999). Skeletal muscle phosphocreatine recovery in exercise-trained humans is dependent on O_2_ availability. J Appl Physiol.

[CR15] Hawkes EZ, Nowicky AV, McConnell AK (2007). Diaphragm and intercostal surface EMG and muscle performance after acute inspiratory muscle loading. Respir Physiol Neurobiol.

[CR16] Hodges PW, Gandevia SC (2000). Changes in intra-abdominal pressure during postural and respiratory activation of the human diaphragm. J Appl Physiol.

[CR17] Hodges PW, Butler JE, McKenzie DK, Gandevia SC (1997). Contraction of the human diaphragm during rapid postural adjustments. J Physiol.

[CR18] Johnson BD, Babcock MA, Suman OE, Dempsey JA (1993). Exercise-induced diaphragmatic fatigue in healthy humans. J Physiol.

[CR19] Katayama K, Yoshitake Y, Watanabe K, Akima H, Ishida K (2010). Muscle deoxygenation during sustained and intermittent isometric exercise in hypoxia. Med Sci Sports Exerc.

[CR20] Katayama K, Iwamoto E, Ishida K, Koike T, Saito M (2012). Inspiratory muscle fatigue increases sympathetic vasomotor outflow and blood pressure during submaximal exercise. Am J Physiol Regul Integr Comp Physiol.

[CR21] Legrand R, Marles A, Prieur F, Lazzari S, Blondel N, Mucci P (2007). Related trends in locomotor and respiratory muscle oxygenation during exercise. Med Sci Sports Exerc.

[CR22] Lin H, Tong TK, Huang C, Nie J, Lu K, Quach B (2007). Specific inspiratory muscle warm-up enhances badminton footwork performance. Appl Physiol Nutr Metab.

[CR23] Lomax M, Grant I, Corbett J (2011). Inspiratory muscle warm-up and inspiratory muscle training: separate and combined effects on intermittent running to exhaustion. J Sports Sci.

[CR24] Miller MR, Hankinson J, Brusasco V, Burgos F, Casaburi R, Coates A, Crapo R, Enright P, van der Grinten CP, Gustafsson P, Jensen R, Johnson DC, MacIntyre N, McKay R, Navajas D, Pedersen OF, Pellegrino R, Viegi G, Wanger J, ATS/ERS Task Force (2005). Standardisation of spirometry. Eur Respir J.

[CR25] Mohr M, Krustrup P, Bangsbo J (2003). Match performance of high-standard soccer players with special reference to development of fatigue. J Sports Sci.

[CR26] Ohya T, Aramaki Y, Kitagawa K (2013). Effect of duration of active or passive recovery on performance and muscle oxygenation during intermittent sprint cycling exercise. Int J Sports Med.

[CR27] Romer LM, McConnell AK (2004). Inter-test reliability for non-invasive measures of respiratory muscle function in healthy humans. Eur J Appl Physiol.

[CR28] Romer LM, Polkey MI (2008). Exercise-induced respiratory muscle fatigue: implications for performance. J Appl Physiol.

[CR29] Romer LM, Miller JD, Haverkamp HC, Pegelow DF, Dempsey JA (2007). Inspiratory muscles do not limit maximal incremental exercise performance in healthy subjects. Respir Physiol Neurobiol.

[CR30] Ross EZ, Nowicky AV, McConnell AK (2007). Influence of acute inspiratory loading upon diaphragm motor-evoked potentials in healthy humans. J Appl Physiol.

[CR31] Tong TK, Fu FH (2006). Effect of specific inspiratory muscle warm-up on intense intermittent run to exhaustion. Eur J Appl Physiol.

[CR32] Volianitis S, McConnell AK, Koutedakis Y, Jones DA (2001). Specific respiratory warm-up improves rowing performance and exertional dyspnea. Med Sci Sports Exerc.

[CR33] Wen AS, Woo MS, Keens TG (1997). How many maneuvers are required to measure maximal inspiratory pressure accurately?. Chest.

[CR34] Wijkstra PJ, van der Mark TW, Boezen M, van Altena R, Postma DS, Koëter GH (1995). Peak inspiratory mouth pressure in healthy subjects and in patients with COPD. Chest.

[CR35] Yaicharoen P, Wallman K, Bishop D, Morton A (2012). The effect of warm up on single and intermittent-sprint performance. J Sports Sci.

